# Design and Optimization of Nanostructured Lipid Carrier Containing Dexamethasone for Ophthalmic Use

**DOI:** 10.3390/pharmaceutics11120679

**Published:** 2019-12-14

**Authors:** Eszter L. Kiss, Szilvia Berkó, Attila Gácsi, Anita Kovács, Gábor Katona, Judit Soós, Erzsébet Csányi, Ilona Gróf, András Harazin, Mária A. Deli, Mária Budai-Szűcs

**Affiliations:** 1 Institute of Pharmaceutical Technology and Regulatory Affairs, Faculty of Pharmacy, University of Szeged, Eötvös u. 6, H-6720 Szeged, Hungary; l.kiss.eszter@pharm.u-szeged.hu (E.L.K.); berkosz@pharm.u-szeged.hu (S.B.); gacsi.attila@pharm.u-szeged.hu (A.G.); anita.kovacs@pharm.u-szeged.hu (A.K.); katona@pharm.u-szeged.hu (G.K.); csanyi@pharm.u-szeged.hu (E.C.); 2 Department of Ophthalmology, Faculty of Medicine, University of Szeged, Korányi Fasor 10-11, H-6720 Szeged, Hungary; juditsoos82@yahoo.com; 3 Institute of Biophysics, Biological Research Centre, Temesvári krt. 62, H-6726 Szeged, Hungary; grof.ilona@brc.hu (I.G.); harazin.andras@brc.hu (A.H.); deli.maria@brc.hu (M.A.D.); 4 Doctoral School of Biology, University of Szeged, Dugonics tér 13, H-6720 Szeged, Hungary; 5 Department of Cell Biology and Molecular Medicine, University of Szeged, Somogyi u. 4, H-6720 Szeged, Hungary

**Keywords:** dexamethasone, nanostructured lipid carrier, factorial design, zeta potential, particle size, entrapment efficiency, human cornea cells toxicity, porcine cornea penetration, Raman mapping

## Abstract

The aim of this study was to perform a preformulation study of dexamethasone (DXM)-loaded nanostructured lipid carriers (NLCs) for ocular use. Lipid screening was applied to find the most suitable solid and liquid lipids and surfactant for the NLC formulation. The visual observation was proved with XRD measurements for the establishment of the soluble state of DXM. Thermoanalytical measurements indicated that the most relevant depression of the crystallinity index could be ensured when using a 7:3 solid lipid:oil ratio. In order to optimize the NLC composition, a 2^3^ full factorial experimental design was used. It was established that each independent factor (lipid, DXM, and surfactant concentration) had a significant effect on the particle size while in the case of entrapment efficiency, the DXM and surfactant concentrations were significant. Lower surfactant and lipid concentrations could be beneficial because the stability and the entrapment efficacy of NLCs were more favorable. The toxicity tests on human cornea cells indicated good ophthalmic tolerability of NLCs. The in vitro drug release study predicted a higher concentration of the solute DXM on the eye surface while the Raman mapping penetration study on the porcine cornea showed a high concentration of nanocarriers in the hydrophylic stroma layer.

## 1. Introduction

Vision significantly acts on quality of life while the effective treatment of ocular diseases presents research & development specialists with a range of challenges. The anatomy, physiology, and biochemistry of the eye create an effective barrier against foreign substances, therefore it is a challenging task to achieve an optimal concentration of drug at the site of action for an appropriate time [1]. The eye has a small absorption surface and has many protection mechanisms, such as solution drainage, lacrimation, blinking, tear turnover, conjunctival absorption, and the relative impermeability of the corneal epithelial membrane. As a result, the most commonly used ophthalmic formulations, eye drops, have a bioavailability of less than 5% [2], and frequent administration is required to provide a therapeutic effect. However, frequent administration could decrease patient compliance and increase the potential of side effects [3].

Topical non-invasive application of ophthalmic preparations is the most advantageous way of drug administration, especially to the anterior segment of the eye. However, as we mentioned, the bioavailability of therapeutic agents is very low in this way. In recent years, nanostructured lipid carriers (NLCs) as lipid nanoparticles have been studied as potential ocular delivery systems. The use of NLCs has many advantages in ophthalmic therapy. NLCs can be alternative delivery systems for microemulsions or liposomes for ophthalmic administration because they can provide small particle sizes (50–400 nm) while containing a lower emulsifier amount [4,5]. Compared with liposomes, higher drug entrapment efficiency can be provided with the application of NLCs due to the decrease in lipid crystallinity [6]. They may enhance corneal penetration and thereby improve bioavailability in a safe, non-invasive, and patient-compatible way [7]. Furthermore, the possible mucoadhesive nature of NLCs may improve their interaction with the cornea membrane, resulting in longer residence times, increased bioavailability, and decreased local side effects. The possibility of scaling up to industrial production is an incontrovertible advantage of these systems.

NLC preparations have been studied for the treatment of various disorders of the eye, such as inflammation, infections, glaucoma, and disorders affecting the posterior segment of the eye [7], where ophthalmic drugs, such as non-steroidal anti-inflammatory drugs (ibuprofen [8], flurbiprofen [9], indomethacin [10]), steroidal anti-inflammatory drugs like triamcinolone acetonide [11], and dexamethasone were incorporated in NLCs [12]. The literature data indicate that NLCs can also be used to treat the anterior and posterior segments of the eye. For the treatment of the anterior segment of the eye, Xiang Li et al. incorporated ibuprofen into NLCs, which resulted in apparent permeability coefficients 1.28 and 1.36 times higher than for the control composition while the AUC of the optimized NLC formulation was 3.99 times higher in the aqueous humor than that of the conventional eye drops [8]. Enhanced transcorneal permeation was achieved with flurbiprofen (FB) loaded NLCs by Gonzales-Mira et al., where NLCs were prepared with stearic acid, castor oil, and Tween^®^ 80, where a central composite rotatable design 2^3^ was used based on three independent variables (oil concentration, Tween^®^ 80 concentration, flurbiprofen concentration). This was a useful tool for optimizing FB-loaded NLC formulations. The developed systems were physically and chemically stable and had high ocular tolerance [9].

The permeation of the therapeutic agents from the eye surface into the posterior segment is very poor, so there is a need for alternatives to more effective invasive pathways, such as intravitreal and subconjunctival injections. These invasive administrations have high risks of infections, bleeding, and loss of vision [7]. For the posterior segment therapy, Balguri et al. formulated indomethacin-based NLCs. NLC formulations showed increased drug load capacity, occlusive effect, and drug delivery into the front and back segments of the eye tissues [10]. 

Corticosteroids are often used in the treatments of various ophthalmic inflammatory diseases, for instance, after complicated eye surgery and corneal transplantation, and for severe uveitis. In many cases, the therapy should be supplemented with systemic corticosteroids or by intraocular or subcutaneous injections. Both frequent eye drops and systemic and/or intraocular supplement therapies can lead to side effects and decreasing patient compliance [13]. Araújo et. al. encapsulated triamcinolone acetonide (TA) into NLCs in order to increase its eye bioavailability. TA is a corticosteroid drug, currently administered by intravitreal injection, for the treatment of a broad spectrum of inflammatory, oedematous, and angiogenic eye diseases. Nanometric (~200 nm), unimodal and negatively charged NLCs were developed to deliver the lipophilic TA through the cornea and the non-cornea to the back segment of the eye. The results showed a quite stable particle size with a low tendency of particle aggregation during storage [11].

The aim of our study was to develop a dexamethasone (DXM)-loaded NLC composition for ocular drug delivery in order to increase the poor water solubility and penetration of DXM, and thus improve the bioavailability of this highly lipophilic drug. In our work, 0.05% and 0.1% DXM concentrations were applied (these two concentrations can be found in the conventional eye drops in the international market, e.g., MAXIDEX^®^ eye drops, suspension with 0.1% DXM; and Maxidex^®^ ophthalmic ointment with 0.05% DXM concentration in phosphate salt). In the first part of our work, lipid screening (solubility study, XRD, DSC measurements, formulation of NLCs) was applied for the following purposes: (1) To find the most suitable solid and liquid lipids and surfactant that are able to dissolve the required dose of DXM in the nano lipid particles; (2) to decrease the crystallinity of the lipids in order to increase the drug loading capacity; and (3) to find the composition that can form a nano system. In the second part of our work, a 2^3^ full factorial design was applied for the final optimization of the NLC compositions, where the zeta potential, particle size, and entrapment efficiency were the optimization parameters used as indicators of the effective and stable nanoparticulate system. The ophthalmic toxicity of the optimized formulations was analyzed by human cornea cells using cell viability measurements. The effect of the optimized NLC formulation on the drug release and penetration of DXM on the cornea was analyzed by carrying out in vitro drug diffusion and ex vivo Raman mapping porcin cornea penetration studies.

## 2. Materials and Methods 

### 2.1. Materials

DXM was purchased from Sigma-Aldrich (St. Louis, MO, USA). Compritol 888ATO (glycerol dibehenate), Apifil (PEG-8 beeswax), and Labrasol (Caprylocaproyl Polyoxyl-8 glycerides) were kindly supplied by Azelis Hungary Ltd. (Budapest, Hungary). Miglyol 812N (capric triglyceride) was provided by Sasol GmbH (Witten, Germany). Kolliphor (or Cremophor) EL (Polyoxyl 35 hydrogenated castor oil, HLB value: 12–14) and Cremophor RH60 (PEG-60 hydrogenated castor oil, HLB value: 15–17) were kindly supplied by BASF SE Chemtrade GmbH (Budapest, Hungary). 

### 2.2. Lipid Screening

For the lipid screening, DXM in two concentrations (0.05 *w*/*w*% and 0.1 *w*/*w*% for the total NLC system) was dissolved in the chosen lipid composition (lipid material or blends). The lipid compositions were heated at 85 °C and DXM was added and mixed with a magnetic stirrer at about 100 rpm (Stuart CB162 magnetic stirrer, Merck, Germany).

During the lipid screening, for the calculation of the DXM concentration in the lipids, 10 *w*/*w*% and 15 *w*/*w*% total lipid contents of the final NLCs were taken as the basis. The investigated solid lipids were Compritol 888 ATO, Apifil; the oils were Miglyol and Labrasol; and the surfactants were Cremophor RH60 and Kolliphor EL.

#### 2.2.1. Visual Observations of DXM Solubility

DXM solubility was investigated visually in the chosen lipid composition (solid lipids alone, liquid lipids alone, and the mixtures of solid and liquid lipids with and without surfactant). The DXM concentrations were 0.3% and 0.7% in the lipid mixture, which corresponded to a 0.05% and 0.10% DXM concentration, calculated with 15% lipid content in the final NLC formulations. The lipid compositions were prepared on the basis of Section 2.2, and the melted lipid compositions (Sol 1–24) were stirred for maximum 8 h at 85 °C. The visual observation of the insoluble DXM particles was executed during these 8 h. 

#### 2.2.2. XRD Analysis of Lipid Mixtures

The solute state of DXM in the solidified lipid composition was analyzed with XRD. The lipid mixtures (LMs) containing solid and liquid lipids in a 9:1 and 7:3 ratio (Table 1) were prepared on the basis of Section 2.2.1, and the melts were cooled to room temperature and investigated after 24 h. 

The XRD measurements were performed with a Bruker D8 Advance diffractometer (Bruker AXS GmbH, Karlsruhe, Germany). The Cu K λI radiation (λ = 1.5406 Å) was used. The samples were scanned from 5° to 40° 2θ at 40 kV and 40 mA. The scanning speed was 0.1°/s, and the step size was 0.010°.

#### 2.2.3. Investigation of Lipid Crystallinity with Differential Scanning Calorimetry (DSC) Measurements

The melting behavior and the crystallinity state (crystallinity index) of lipid mixtures and DXM-containing lipid mixtures were examined with DSC (Mettler-Toledo 821e DSC instrument (Mettler-ToledoGmbH, Greifensee, Switzerland). The sample preparation is described in Section 2.2.2. Samples of 5 mg were weighed into aluminum pans, and sealed, and an empty aluminum pan was used as a reference. The scanning was performed from 10 to 100 °C at the heating rate of 5 K/min. The nitrogen flush (50 mL/min) was constant. The sample compositions are listed in Table 1.

From the results of the DSC measurements, the crystallinity index (CI%) of the lipid compositions was calculated with Equation (1) [14]:

(1) $$CI\;\%=\;\frac{\Delta H_{bulk\;material}\times solid\;lipid\;ratio}{\Delta H_{solid\;lipid}}\times 100.$$

#### 2.2.4. Investigation of NLC Formulations

The test NLC systems (T-NLCs) were prepared with the ultrasonication method. The solid lipid, liquid lipid, and surfactant were melted with a heating magnetic stirrer at 85 °C. Then, DXM was added to the melted lipid mixture and mixed in it. The pure water was heated at 85 °C and the two phases were mixed and ultrasonicated (one cycle, 70% amplitude, 10 min) with a Hielscher UP200S ultrasonic homogenizer (Hielscher Ultrasonics GmbH, Germany). The NLC systems were cooled in an ice bath. The drug-free test NLC compositions (T-NLC) are in Table 2.

T-NLCs were analyzed by particle size and particle size distribution measurements using laser diffraction, Mastersizer 2000 tool (Malvern Instruments, United Kingdom). The d(0.1), d(0.5) and d(0.9) were assessed. The measuring medium was purified water with a refractive index (RI) of 1.33. The RI of the samples was 1.36 and the imaginary RI was 0.01. The span value can be calculated with the following Equation (2):

(2) $$Span\;=\;\frac{d\left(0.9\right)-d\left(0.1\right)}{d\left(0.5\right)}.$$

### 2.3. Experimental Design

A 2^3^ full factorial design was made, which is suitable for the investigation of the linear response surface and for generating a first order polynomial model (Equation (3)). The model describes the principal effects and interaction among the identified variables:

(3) $$y=a_{0}+a_{1}x_{1}+a_{2}x_{2}+a_{3}x_{3}+a_{12}x_{1}x_{2}+a_{23}x_{2}x_{3}+a_{13}x_{1}x_{3},$$

where *a*_0_ is the intercept and *a*_1,2,3_ are the regression coefficient values. *x*_1_*, x*_2_, and *x*_3_ correspond to factors A, B, and C, respectively.

The samples were prepared by the ultrasonication method to evaluate the effects of the three DXM-NLC formulation factors. The factors were A (lipid concentration), B (DXM concentration), and C (surfactant concentration). The optimization parameters (dependent factors) were the particle size (hydrodynamic diameter, Z_ave_), Zeta potential (ZP), polydispersity index (PI), and entrapment efficacy (EE%). The chosen factors were examined at two levels (+1 and −1). Statistical data analysis was performed using Statistica for Windows, version 10. The compositions of the factorial design can be found in Table 3.

#### 2.3.1. Particle Size and Zeta Potential of NLCs

The Z_ave_, ZP, and PI of NLCs 1–8 were investigated by the Zetasizer Nano ZS instrument (Malvern Instruments, Malvern, UK) with DTS 1070 folded capillary cell at 25 °C. The applied medium was purified water. The refractive index was 1.3553. Three parallel measurements were made, and 6-fold dilution was used. 

#### 2.3.2. Entrapment Efficacy of NLCs

The EE% of NLCs was determined with an indirect method. The clear aqueous phase of the NLCs was separated by centrifugation in Vivaspin 15R 5000 MWCO Hydrosart tubes (Sartorius, Stonehouse, UK) with Hermle Z323K, (HERMLE Labortechnik GmbH, Wehingen, Germany). Centrifugation was performed at 5000 rpm (9000 rcf), for 30 min at 4 °C. 

The DXM content of the filtered solution was examined by high-performance liquid chromatography (HPLC) (Shimadzu Nexera X2 UHPLC, Kyoto, Japan), which was equipped with a C18 reverse-phase column (Phenomenex Kinetex EVO C18, Phenomenex, Torrance, CA, USA) with dimensions of 1.7 µm, 100 Å, 100 × 2.1 mm. The mobile phase was water:acetonitrile 75:25 in isocratic elution, and the detection was made at 240 nm. The injection volume was 5 µL and the flow rate was 0.5 mL/min. The column temperature was 40 °C. The time of analysis was 6 min and the retention time for DXM was 3.5 min.

The following equation was used to calculate EE% (Equation (4)):

(4) $$EE\;\%=\;\frac{W_{initial\;drug}-W_{free\;drug}}{W_{initial\;drug}}*100.$$

### 2.4. In Vitro Drug Release Study

Considering the results of the factorial experimental design, four NLC compositions were selected for the diffusion study (Table 3; NLC1,3,5,7). To investigate the in vitro drug release, the dialysis bag method was used [15]. Firstly, 200 µL of the samples (NLC1,3,5,7) were closed in a Spectra/Por^®^ 4 dialysis membrane (Spectrum Laboratories, Inc., Rancho Dominguez, CA, USA), with Spectra/Por^®^ Closures (Spectrum Laboratories, Inc.). From each composition of NLCs, 3 replicates were used and placed into 20 mL of phosphate-buffered saline (PBS) (PBS of pH = 7.4 was prepared by dissolving 8 g dm^−3^ NaCl, 0.2 g dm^−3^ KCl, 1.44 g dm^−3^ Na_2_HPO_4_.2H_2_O, and 0.12 g dm^−3^ KH_2_PO_4_ in distilled water, the pH being adjusted with 0.1 M HCl). The sink condition was ensured during the experiments. The systems were held at 35 °C to mimic in vivo conditions and stirred at 370 rpm with a magnetic stirrer. During the diffusion study, seven sampling times were used (0.5, 1, 2, 3, 4, 5, and 6 h) by removing 1 mL from the acceptor phase and replacing it with PBS to maintain sink conditions. As a reference preparation, a DXM suspension (0.1 *w*/*w*%) was formulated (d(0.5) = 7.8 µm measured by Mastersizer 2000). The diffused amounts of DXM were analyzed by HPLC (the HPLC method is described in Section 2.3.2).

### 2.5. Cell Viability Measurements

For the cell culture experiments, all reagents were purchased from Sigma-Aldrich Kft., Hungary, unless otherwise indicated. Human corneal epithelial cells (HCE-T; RCB 2280; RIKEN BRC, Tsukuba, Japan) were immortalized by transfection with a recombinant SV40-adenovirus vector, established and characterized by Araki-Sasaki [16]. The cells were grown in Dulbecco’s modified eagle’s medium/F-12 (Gibco, Life Technologies, Carlsbad, CA, USA) supplemented with 10% fetal bovine serum (Gibco, Life Technologies, Carlsbad, CA, USA), 0.5% dimethyl sulfoxide (DMSO), 5 µg/mL recombinant human insulin, and 10 ng/mL recombinant human epidermal growth factor (EGF) in a humidified incubator with 5% CO_2_ at 37 °C. All plastic surfaces were coated with 0.05% rat tail collagen in sterile distilled water before cell seeding in culture dishes. The culture medium was changed every second day. When cells reached approximately 80% to 90% confluence in the dish, they were subcultured with 0.05% trypsin/EDTA solution. For the MTT cytotoxicity assays, cells were cultured in 96-well plates (Corning Life Sciences, Tewksbury, MA, USA) for 3 days. 

The metabolic activity and viability of the cells were used to measure by 3-(4,5-Dimethyltiazol-2-yl)-2,5-diphenyltetrazolium bromide (MTT) dye conversion. The yellow MTT is enzymatically converted by viable cells to purple formazan crystals. A decrease in formazan production reflects cellular damage. Confluent HCE-T cultures were treated for 6 h. To prepare dexamethasone and Cremophor RH60 solutions, DMSO was used to dissolve the reagents. Treatment solutions were prepared in cell culture medium, and the final concentrations were: 0.1 mg/mL for dexamethasone, and 2.5 and 5 mg/mL for Cremophor RH60. The different formulations were diluted 10 and 100 times in cell culture medium. After removing the treatment solutions, the cells were incubated with 0.5 mg/mL MTT solution for 1 h in a CO_2_ incubator. The amount of formazan produced by the cells was dissolved in DMSO and determined by measuring the absorbance at the 570-nm wavelength with a microplate reader (Fluostar Optima; BMG Labtechnologies, Ortenberg, Germany). Cell viability was calculated as the percentage of dye conversion by non-treated cells. Triton X-100 (10 mg/mL) (TX-100) detergent was used as a toxicity control. The investigated samples were NLC3, NLC7, Cremophor RH60, and DXM.

### 2.6. Porcine Corneal Penetration Study of NLCs

The porcine conjunctiva was obtained from a slaughterhouse and stored at −20 °C until the measurement. The porcine cornea was placed onto a sterile cotton wool bed wetted with physiological saline solution and the cornea was surrounded with a Teflon ring in order to avoid the flow of the eye drop toward the conjunctiva. The cornea was instilled with 250 µL of NLC7 every 30 min, and the formulation was removed just before the next instillation. The length of the treatment was six hours. The system was thermostated at 35 °C. The treated cornea was frozen and sectioned (20 µm thick cross-sections) onto aluminum-coated slides using a Leica CM1950 cryostat (Leica Biosystems GmbH, Wetzlar, Germany). As a reference, an untreated porcine corneal section was used. Raman microscopy measurements were performed with the Thermo Scientific DXR Raman microscope (Thermo Fisher Scientifc, Waltham, MA, USA). A laser light of a 780-nm wavelength was used, and its maximum power was 24 mW. The microscopic lenses used for measurements were magnified 50× and the aperture was 25 mm. The examined chemical mapping area was 150 × 1200 µm; the step size was vertically and horizontally 50 µm. A total of 72 spectra were recorded, 16 images were collected for each spectrum, and the exposure time was 5 s. The instrument operation and the evaluation of the measurements were carried out by OMNIC for Dispersive Raman 8.2 software package (Thermo Fisher Scientifc). The characteristic peaks of the DXM spectrum coincided with the characteristic peaks of the corneal tissue, however, the profiled map showed a detectable signal of NLC7. For this reason, profiling of the Raman map was performed on the spectrum of the full preparation during the evaluation. 

### 2.7. Statistical Analysis

The drug release results were statistically analyzed with GraphPad Prism version 5 software. Two-way ANOVA analysis was applied with Bonferroni post-tests. For the MTT assay, the values were compared using ANOVA followed by Dunett’s test. The values are expressed as means ± standard deviation (SD). A level of *p* ≤ 0.05 was taken as significant, *p* ≤ 0.01 as very significant, and *p* ≤ 0.001 as highly significant.

## 3. Results and Discussion

### 3.1. Lipid Screening

NLCs contain solid lipid, liquid lipid, and surfactant in an aqueous medium. The EE% of lipid nanoparticles can be improved by reducing the crystallinity of solid lipids. The liquid lipid decreases the crystallinity index of solid lipids in blends, and thus the crystallinity of lipid nanoparticles. The application of surfactants can also decrease the crystallinity index of lipid particles and help to form a nano emulsion system during the formulation, and their presence at the water–lipid interface can ensure the stability of nanoparticles. It is important to note that the surfactant can cause irritation and toxicity, especially on the eye. Therefore, in the case of NLC systems, a lower surfactant concentration may be sufficient. In our work, the selection criteria of the components were a low irritation potential, eye tolerability, and suitability for NLC formulation based on the literature background. Tayel et al. (2013) examined a Cremophor EL-containing formulation on in vivo rabbit eyes and it provoked no histopathological alteration in the investigated tissues, including the cornea, iris, retina, and sclera [17].

The lipid screening focused on three potential critical factors that may have an effect on the performance of NLCs. The first was to find lipid compositions that are able to solve the applied dose of DXM. In the first part of the solubility study, a cost-effective method of visual observation was used to detect insoluble DXM crystals in the different combinations of lipid melts. In the second part of this study, the solubility of DXM in the solidified lipids was analyzed with XRD measurements. The second critical factor is the crystallinity of the applied lipids because their crystallinity index (CI%) may have an effect on the drug loading capacity of NLCs. This factor was investigated with DSC measurements. The third critical factor is the ability of the lipid–surfactant blends to form an NLC formulation, therefore blank (drug-free) test NLCs (T-NLCs) were formulated and analyzed by laser diffraction.

#### 3.1.1. Visual Observations of DXM Solubility

In this part of the solubility study, a fast screening method of the visual observation of different combinations of lipid melts, including solid lipids, liquid lipids alone, and their mixtures with and without surfactant (Table 4), was applied. When insoluble particles were observed in the thin layer of the melted lipid, it was considered as not solved.

The active pharmaceutical ingredient (API) could not be dissolved in the chosen lipids with the exception Labrasol, which was able to dissolve DXM in both concentrations. In the Compritol and Kolliphor RH60 mixtures, the API in a low concentration (0.3 *w*/*w*%) could be dissolved completely while the dissolution of a high concentration (0.7 *w*/*w*%) of DXM was partial. The same tendency was seen in the case of the Kolliphor EL and Compritol 888 ATO mixture. In the Apifil and surfactant mixtures, the API was dissolved in both concentrations. In this solubility study, there was no difference between the two solid lipids (Compritol 888 ATO, Apifil). Compared to Miglyol, Labrasol was much more effective at dissolving API. In the case of solid lipid (Compritol 888 ATO), oils (Miglyol, Labrasol), and surfactant (Cremophor RH60, Kolliphor EL)-containing mixtures, DXM was dissolved in almost all cases, except for Sol-21 and Sol-22, where the mixtures were made with Apifil, Miglyol, and Cremophor RH60(Sol-21)/Kolliphor EL(Sol-22).

#### 3.1.2. XRD Analysis of Lipid Mixtures

The visual observation revealed that the lipid mixtures containing solid, liquid lipids, and surfactant were able to solve the required DXM amounts. XRD is a technique used to investigate the solid state of a drug, giving information about its amorphous or crystalline nature. The disappearance of the crystalline peaks mainly indicates the amorphous form of the drug within the lipid matrix and/or its molecularly dispersion in the lipid. In this section, 7:3 and 9:1 ratios of the solid and liquid lipids were evaluated. 

**Figure 1:**
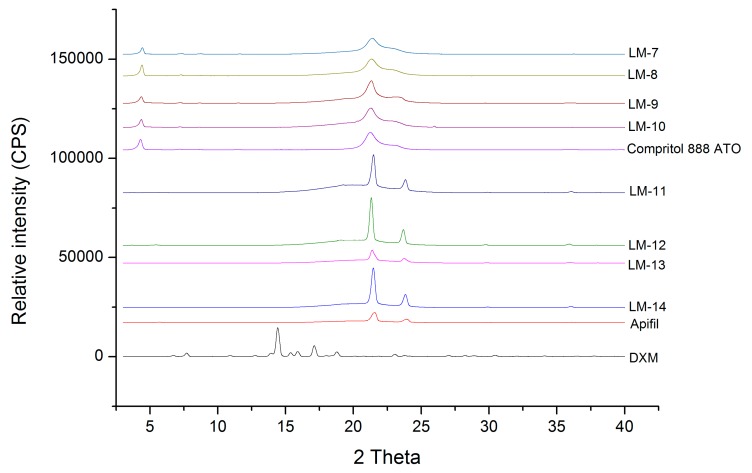
X-ray diffractograms of DXM, Apifil, Compritol 888 ATO, and various lipid mixtures with DXM.

During the XRD measurements, the presence of the diffraction peaks of crystalline DXM was investigated. The X-ray diffraction pattern of dexamethasone showed major peaks at 2θ = 6.1, 9.1, 10.9, 12.86, 14.66, 15.4, and 17.02 in our measurements, similar to the investigation by Ali et al. (2013) [18]. The fact that the characteristic peaks of crystalline DXM are not detectable can prove its amorphous form and/or molecular dispersion in all the lipid matrices and it can be concluded that the ratio of the solid and liquid lipids did not influence it (Figure 1).

On the other hand, the characteristic peaks of the solid lipids (the characteristics peaks of Apifil are 2θ = 21.51, 39.32, 36.06, 5.67 and the main peaks of Compritol 888 ATO are 2θ = 4.27, 21.25, 22.97) can be found in the lipid mixtures, indicating the presence of some crystalline solid lipid in all cases. The amount of crystalline solid lipid is detailed with the DSC measurements in Section 3.1.3.

#### 3.1.3. Investigation of Lipid Crystallinity with DSC Measurements

Lipid crystallization plays a very important role in the performance of NLC carriers because it has a great influence on the drug loading capacity of lipid particles and the drug release from them.

It must be considered that the application of liquid lipids/oily components can decrease the melting point of the lipid nanoparticle, which can result in easy diffusion of the API from the nanocarriers. From this point of view, the melting behavior of the lipid mixture can be an important factor because it can predict the permanence of the amount of the incorporated drug in the nanocarriers during storage and can have an influence on the evaluation of the drug effect. 

As it was mentioned, a strategy to decrease crystallinity is the application of liquid lipids. In our work, two oil and solid lipid ratios (7:3 and 9:1) were evaluated. On the other hand, emulsifiers can also have an effect on the crystallinity, so two emulsifier types (Cremophor RH60 and Kolliphor EL) in the same concentration were also investigated in this section.

Based on the results, it can be seen that Cremophor RH60 decreased the melting point of the lipid mixture in the case of the 7:3 solid lipid:oil ratio (samples LM-3 and LM-7). In the case of Kolliphor EL (LM-4,5) and the 9:1 solid lipid:oil ratio, this decrease in the melting point was not experienced.

The two emulsifiers (Cremophor RH60 and Kolliphor EL) reduced the crystallinity index, but there was no remarkable difference between them (Table 5). The higher liquid lipid concentration resulted in the most relevant CI% depression. The presence of the API (samples LM 7–10) can also change the CI%, but just in the case of the 7:3 solid lipid:oil ratio. In this latter case, the slight increase in CI% (LM-7: 23.26 vs. LM-3: 20.95, and LM-8: 23.11 vs. LM-4: 18.33) could be observed.

Thanks to the favorable CI% depression effect of the 7:3 solid lipid:oil ratio (with minimal melting point depression), this ratio was applied for further optimization.

#### 3.1.4. Investigation of NLC Formulations

In order to clarify the ability of the chosen lipid mixtures to form a nanosystem, drug-free test NLCs (T-NLCs) were made. To characterize the NLC systems, the following factors were measured using the laser diffraction method: d(0.1), d(0.5), d(0.9), and span values. 

On the basis of Equation (2), the lower span value (at about 1 or less) indicates a more monodispersed distribution, thus better stability for NLC systems. The span value was high for T-NLCs 1, 5, 6, and 11. The T-NLCs made with Apifil (T-NLCs 1, 2, 5, 6, and 11) had higher span values than Compritol 888 ATO-containing NLCs (Table 6). The mixtures containing Labrasol as liquid lipid did not form NLC systems (T-NLCs 10–16); the only exception was T-NLC 11, where nanosized particles could be detected but the span value was very high (2.776) (Table 6). As a consequence, Labrasol as an oil component was excluded from further measurements.

The lowest span value was found for T-NLC 3, which was made with Compritol 888 ATO, Miglyol, and Cremophor RH60. So, components of T-NLC 3, such as Compritol 888 ATO, Miglyol 812N, and Cremophor RH60, were chosen for further measurements and formulation optimization.

### 3.2. Optimization of DXM-Loaded NLCs with Factorial Experimental Design

During the lipid screening, we could conclude that the optimal components for NLC formulation are Compritol 888 ATO, Miglyol 812N, and Cremophor RH60 with a 7:3 solid lipid:liquid lipid ratio. To evaluate the effect of the component concentration and find the optimal ratio, a factorial experimental design was used. With the application of a 2^3^ full factorial design, over the optimal ratio, information about the interactions of factors can also be provided. Three formulation parameters of DXM-NLC were chosen as independent factors (the concentration of the emulsifier, the drug, and the total lipid). As for the optimization parameters (dependent factors), the particle size (Z_ave_), zeta potential (ZP), polydispersity index (PDI), and entrapment efficacy (EE%) of the NLC systems were chosen. 

As it was mentioned above, the emulsifier is essential for the formulation of NLC systems, but it can be the main irritating and toxic component of the formulations. For this reason, an effort should be made to minimize the amount of surfactant. In the factorial design, the two levels of emulsifier concentration were 5% and 2.5%. As the high lipid concentration can make it possible to solve more API, 10% and 15% total lipid contents were evaluated with 0.05% and 0.1% total DXM concentrations during our experiments. 

EE% provides information about the amount of DXM, which can be incorporated into the lipid matrix of the NLC. 

ZP is a key indicator of the stability of colloid dispersions. The magnitude of ZP indicates the degree of electrostatic repulsion between adjacent, similarly charged particles in the dispersion. For molecules and particles that are small enough, the high absolute value of ZP provides stability, and these dispersions have moderate aggregation. If the absolute value of ZP is low, the attractive forces may exceed this barrier and the dispersion may break and flocculate. Thus, colloids with a high ZP (negative or positive) are electrically stabilized while colloids with low ZP tend to coagulate or flocculate [19,20]. In the case of an electrostatic and steric stabilized system, about ZP ± 20 mV is advised [7].

PDI is used to estimate the average uniformity of a dispersed solution, and larger PDI values correspond to a larger size distribution in the dispersed sample. PDI can also indicate nanoparticle aggregation and the stability of the dispersed system. A sample is considered monodispersed when the PDI value is less than 0.1. The low PDI (0.1–0.25) shows a narrow size distribution [7] while PDI over 0.5 indicates a wide size distribution, resulting in more polydispersity [21,22]. 

As indicators of the stability of NLCs, the optimization parameters were measured after preparation (after 24 h), and also after 1 month (except for EE%) (Table 7). On the basis of the results, the particle size of the NLC systems was less than 300 nm and the PDIs were less than 0.2, which means a narrow size distribution in all cases. The smallest particles and the narrowest size distributions were detected in the case of NLCs 5 and 7, where the total lipid concentration was low (10 *w*/*w*%) and the surfactant concentration was high (5 *w*/*w*%). The ZP values changed between 8 and 15, which corresponds to the literature data, where the ZP of NLC prepared with the Cremophor-type emulsifier was about −12 mV [23]. These values, the low particle size, and the narrow size distribution can help to retain the stability of the nano systems. The EE% of the nano lipids was very high, over 87%, indicating the good drug loading capacity of the nano lipid carriers. EE% was better in the NLCs containing lower surfactant concentrations (NLCs 1–4), which may be explained by the appearance of the redundant surfactant in the aqueous phase, which can dissolve DXM in the aqueous phase in a higher amount.

When the samples were measured after 1 month, in two compositions (NLCs 4 and 6), the aggregation of the nano lipid particles resulted in the formation of a lipid cake on the top of the container, therefore none of the tested parameters could be measured in these cases. In the case of the homogenous samples, it could be observed that the PDI values remained within the narrow size. The absolute ZP values (if it was applicable) increased with storage time (after 1 month), which means the aqueous–lipid interface changed and became more stable after 1 month.

A mathematical model was used to analyze the single and combined effect of the factors. The coefficients in the equations describe the size and direction (negative: Inversely proportional; or positive: Directly proportional) of the relationship between a term in the model and the response variable [24]. The mathematical model is shown in the following equations (Equations (5)–(8)) of the response surfaces:

EE% = 91.7 + 0.03 A − 1.39 B − 1.85 C − 0.20 AB − 0.28 AC − 0.69 BC,(5)

Z_ave_ (d.nm) = 171.38 + 30.82 A − 1.80 B − 52.12 C + 2.18 AB − 3.96 AC + 2.80 BC, (6)

PDI = 0.16 + 0.02 A + 0.00 B − 0.01 C + 0.01 AB + 0.01 AC + 0.01 BC,(7)

ZP (mV) = −11.15 − 0.58 A + 1.02 B + 2.11 C − 0.27 AB + 0.20 AC + 0.07 BC,(8)

where factor A is the lipid concentration, B is the DXM concentration, and C is the surfactant concentration. 

**Figure 2:**
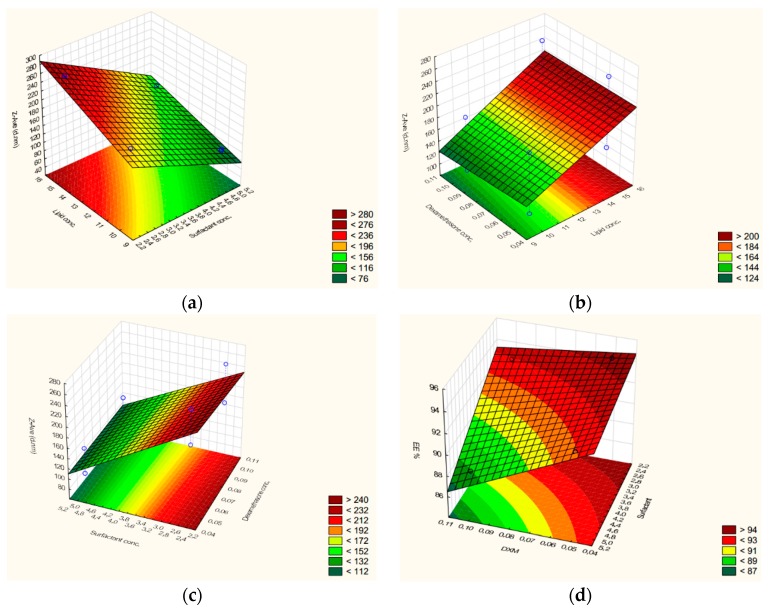
The measured Z_ave_ as a function of lipid concentration, surfactant concentration, and dexamethasone concentration (**a**–**c**). Entrapment efficacy as a function of DXM and surfactant concentration (**d**).

The results suggest that EE% depends on DXM and the surfactant concentration (inversely proportional) as well (Figure 2d). The combined effect of factors is also inversely related to EE%. On the basis of the coefficient value, the changes of entrapment efficiency are significant (higher coefficient values) with the DXM concentration and surfactant concentration while the combined effect of the factors is not remarkable (lower coefficient values). The mathematical model demonstrated that the lipid content does not influence EE% (very low coefficient value), so in this total lipid concentration range (10–15%), the drug loading capacity of NLCs cannot improve by increasing this concentration.

Z_ave_ is also affected by lipid (directly proportional), DXM, and surfactant concentration (inversely proportional) (Figure 2a–c), but the amount of surfactant showed the most remarkable impact. The combined effect of factors is less expressed as it was observed in the case of EE%, too. The increase of the surfactant amount can be beneficial for particle size reduction, but as we could conclude from Equation (4), it can deteriorate the drug loading capacity (lower EE% values).

Concerning PDI, very low coefficient values were calculated. In this case, the preparation technique and/or type of composition may have a more relevant influence on it, but these parameters were constant in our current experiments.

ZP is influenced by DXM, surfactant (directly related), and lipid concentration (inversely related). As it was concluded in the case of EE% and Z_ave_, the combined effect of factors does not have a considerable effect on it. The most remarkable effect can be observed in the case of the surfactant, which means the increase in the surfactant concentration can improve the stability of NLCs (higher absolute ZP value), but higher values were found to reduce EE%. 

As a conclusion, the surfactant concentration has the most remarkable effect on the stability parameter (Z_ave_ and ZP) of NLCs; an increased amount can be favorable, but we must bear in mind that its higher concentration can result in a lower drug loading capacity and potential irritation and toxicity.

### 3.3. In Vitro Drug Release Study

In order to compare our optimized NLCs with the conventional suspension form, in vitro drug release studies were performed. On the basis of the results of our factorial experimental design, the four most relevant compositions were chosen to be compared with 0.1% DXM suspension. Two of the chosen compositions (NLC3 and 7) contained the same concentration of DXM (0.10%) as in the case of the suspension while two of them (NLC1 and 5) contained half the amount (0.05%) of that. The drug release profile of the formulations is shown in Table 8. 

**Figure 3:**
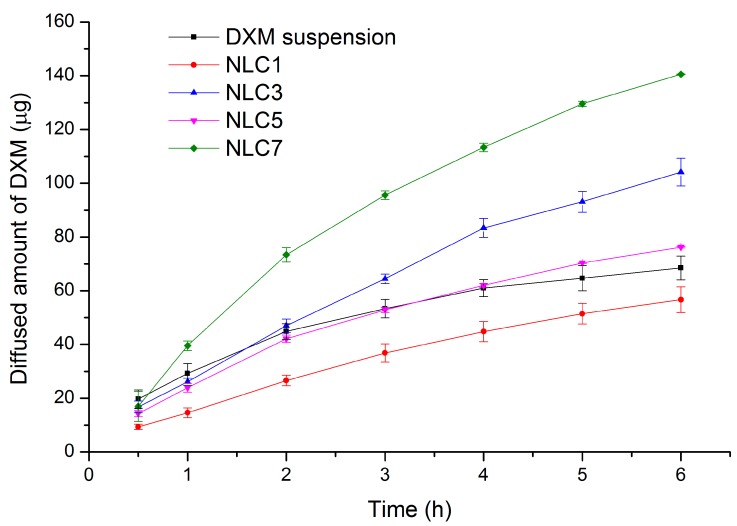
In vitro drug release from NLC formulations (NLC1, 3, 5, 7) and DXM suspension.

The mathematical modelling of the in vitro release data showed the DXM release from NLCs and suspension followed Higuchi release kinetics (R^2^ is higher than 0.99 for NLCs, and higher than 0.98 for suspension). This type of kinetics indicates a diffusion-controlled release from the systems (Table 8). It could be clearly seen the NLCs containing 0.10% DXM showed a remarkable drug release, at 6 h. The amounts of the released drug from NLC7, NLC3, and the DXM suspension were 140, 104, and 68 µg, respectively (Figure 3). These results can predict a 100% increase in drug release using NLC7. When we consider the NLCs containing 0.05% DXM, the NLC-1 composition displayed a lower amount of the released drug at each time point while the NLC5 composition was similar to the suspension form (no significant differences during the drug release, except at 6 h, where a significantly higher drug amount was detected in the case of NLC5). This finding can also confirm that the NLC composition can increase the amount of the released drug.

### 3.4. Cell Viability Assay

In order to analyze the possible toxicity of the formulated NLCs, a human cornea cell viability study was applied, where the most relevant compositions (NLC3 and 7) based on the drug release study, the emulsifier (Cremophor RH60), and DXM were investigated [4].

**Figure 4:**
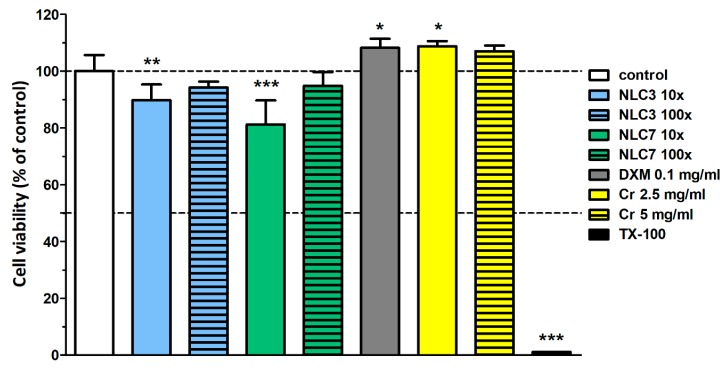
Toxicity of NLC3 and NLC7 formulations, dexamethasone, and Cremophor RH60 on human HCE-T cornea epithelial cells measured by MTT assay after the 6-h treatment. Values are expressed as percentage viability compared to the non-treated group and presented as means ± SD (n = 6–10). Statistical analysis: One-way ANOVA followed by Dunnett’s test. (* *p* ≤ 0.05 significant; ** *p* ≤ 0.01 very significant; and *** *p* ≤ 0.001 highly significant difference from control).

During the colorimetric assay, NLC3 and NLC7 formulations at 10× dilution slightly decreased the viability of the HCE-T cells by 10% and 19%, respectively, after the 6-h treatment. The 100× dilutions did not cause any significant alteration in the cell index, indicating no cell damage (Figure 4). Cremophor RH60 did not reduce the cell viability of the epithelial cells at either the 2.5 or 5 mg/mL concentration for 6 h (Figure 4). These results are in accordance with our previous studies, where concentrations of Cremophor RH40 less than 10 mg/mL were non-toxic for different cultured epithelial cells [25,26].

### 3.5. Porcine Corneal Penetration Study of NLCs 

Topically applied ophthalmic formulations are faced with a number of elimination mechanisms and barriers that reduce the bioavailability of the active ingredients. After instillation, tear production slightly increases in order to wash away the foreign material from the surface of the eye. Following this, through the nasolacrimal sac, the material washed out can enter into the systemic circulation, increasing the risk of potential side effects. In addition, a part of the active agent can also be eliminated via the highly vascularized conjunctiva, further reducing the possibility of its penetration into the deeper ocular tissue layers [27]. The main absorption way of the topically applied drugs is through the cornea, which has a remarkable barrier function. The cornea consists of three main layers: The epithelium (next to the conjunctiva), the stroma (the middle layer), and the endothelium (the inner layer). The epithelium and the endothelium are lipid-rich layers that limit the absorption of hydrophilic drugs while the stroma as an aqueous layer limits the permeation of lipophilic substances. In addition, both the conjunctiva and the corneal epithelium have tight junctions that limit the absorption of substances (regardless of their physicochemical properties) by paracellular permeation, further reducing the bioavailability [27,28].

DXM as a lipophilic drug is considered to be able to penetrate through the epithelial layer, but the stromal layer of the cornea is a significant barrier for this molecule. When DXM is present in a suspension form, a part of it is removed from the ocular surface due to the elimination mechanisms following administration. A part of the suspended lipophilic drug might be soluble in the lipophilic layer of the tear film and can be present in higher concentrations than in the aqueous layer of the tear film. It may penetrate into the lipophilic epithelium but is supposed to poorly penetrate across the hydrophilic stromal layer. However, when NLC is used, it is probable that the better distribution and lower mechanical irritation (less foreign material sensation) of the nano-sized material can result in decreased elimination. The active ingredient will also be present in the lipophilic layer of the tear film as well as in the lipophilic epithelial layer, like in the case of the conventional suspension form. However, the nanocarrier can also pass through the hydrophilic stromal layer and can be distributed in it due to its small size and emulsifier content. 

In order to follow the penetration of nanocarriers through the cornea, in our study, semi-quantitative Raman mapping was applied, where porcine cornea was treated with NLC formulation, and the penetration depth was analyzed.

Based on the results of the in vitro drug release study, NLC7 was chosen for the penetration study.

**Figure 5:**
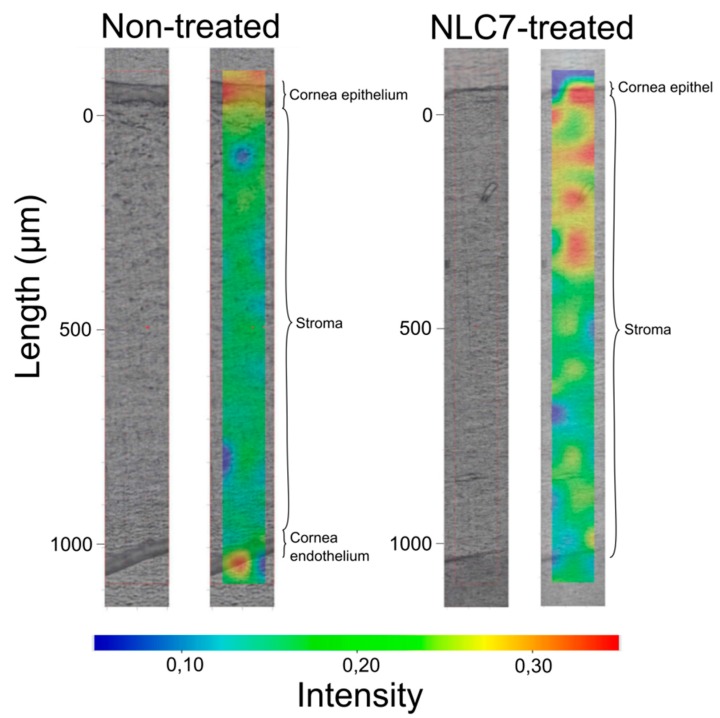
Raman correlation maps of the distribution of NLC7 in the porcine cornea specimen compared with the non-treated cornea specimen.

NLC7-treated porcine cornea specimen was compared with non-treated cornea specimen using Raman correlation mapping. The spectrum of NLC7, containing DXM as well, was used for the cornea distribution correlation maps. Figure 5 shows a high Raman intensity on the top of the cornea specimens in both cases (non-treated, treated), which can correspond to the high lipid content of the epithelial layer and the surface of the tear film. In contrast, the NLC7-treated cornea has a higher Raman intensity for the lipid components in the 200–400 µm depth of the cornea, the depth of which corresponds to the stroma layer. This observation means the nanocarriers entered and enriched in the hydrophilic part of the cornea, which is considered as a main limiting barrier for hydrophobic materials. Based on these results, the penetration of DXM through the cornea might be achieved by using nano lipid carriers.

## 4. Conclusions

Successful formulation of non-toxic potential ophthalmic NLC systems was carried out with a narrow size distribution, high DXM entrapment efficacy, and improved penetration. The chosen components were Compritol 888 ATO, Miglyol 812N, and Cremophor RH60. We propose the use of low levels of surfactant concentration and lipid concentration (e.g., 2.5% for surfactant and 10% for total lipid) because then the critical stability parameters (Z_ave_, ZP, PDI) and drug loading capacity (EE%) are suitable while the emulsifier concentration can remain at low levels.

## Figures and Tables

**Table 1:** The composition of lipid mixtures (LMs) for XRD and DSC measurements in *w*/*w*%.

Sample	Compritol 888 ATO	Apifil	Labrasol	Miglyol 812N	Kolliphor EL	Cremophor RH60	DXM
LM-1	70.00	-	-	30.00	-	-	-
LM-2	90.00	-	-	10.00	-	-	-
LM-3	46.67	-	-	20.00	-	33.33	-
LM-4	46.67	-	-	20.00	33.33	-	-
LM-5	60.00	-	-	6.67	33.33	-	-
LM-6	60.00	-	-	6.67	-	33.33	-
LM-7	46.67	-	-	20.00	-	33.33	0.33
LM-8	46.67	-	-	20.00	33.33	-	0.33
LM-9	60.00	-	-	6.67	33.33	-	0.33
LM-10	60.00	-	-	6.67	-	33.33	0.33
LM-11	-	46.67	20.00	-	-	33.33	0.33
LM-12	-	60.00	6.67	-	-	33.33	0.33
LM-13	-	46.67	-	20.00	-	33.33	0.33
LM-14	-	60.00	-	6.67	-	33.33	0.33

**Table 2:** The composition of drug-free test NLCs (T-NLC) in *w*/*w*%.

Sample	Compritol 888 ATO	Apifil	Miglyol 812N	Labrasol	Kolliphor EL	Cremophor RH60	Purified Water
T-NLC1	-	10.5	4.5	-	5.0	-	80.0
T-NLC2	-	10.5	4.5	-	-	5.0	80.0
T-NLC3	10.5	-	4.5	-	-	5.0	80.0
T-NLC4	10.5	-	4.5	-	5.0	-	80.0
T-NLC5	-	7.0	3.0	-	5.0	-	85.0
T-NLC6	-	7.0	3.0	-	-	5.0	85.0
T-NLC7	7.0	-	3.0	-	5.0	-	85.0
T-NLC8	7.0	-	3.0	-	-	5.0	85.0
T-NLC9	10.5	-	-	4.5	-	5.0	80.0
T-NLC10	10.5	-	-	4.5	5.0	-	80.0
T-NLC11	-	10.5	-	4.5	-	5.0	80.0
T-NLC12	-	10.5	-	4.5	5.0	-	80.0
T-NLC13	-	7.0	-	3.0	5.0	-	85.0
T-NLC14	-	7.0	-	3.0	-	5.0	85.0
T-NLC15	7.0	-	-	3.0	5.0	-	85.0
T-NLC16	7.0	-	-	3.0	-	5.0	85.0

**Table 3:** The compositions of factorial design.

Sample	Lipid Concentration (*w*/*w*%)	DXM Concentration (*w*/*w*%)	Surfactant Concentration (*w*/*w*%)
NLC1	10	0.05	2.5
NLC2	15	0.05	2.5
NLC3	10	0.10	2.5
NLC4	15	0.10	2.5
NLC5	10	0.05	5.0
NLC6	15	0.05	5.0
NLC7	10	0.10	5.0
NLC8	15	0.10	5.0

**Table 4:** The composition of lipid matrices in the solubility study.

Sample	Compritol 888 ATO	Apifil	Miglyol 812N	Labrasol	Kolliphor EL	Cremophor RH60	DXM	Solubility
Sol-1	89.7						0.3	-
Sol-2	89.3						0.7	-
Sol-3		89.7					0.3	-
Sol-4		89.3					0.7	-
Sol-5			89.7				0.3	-
Sol-6			89.3				0.7	-
Sol-7				89.7			0.3	+
Sol-8				89.3			0.7	+
Sol-9	66.3					33.3	0.3	+
Sol-10	66.0					33.3	0.7	-
Sol-11	66.3				33.3		0.3	+
Sol-12	66.0				33.3		0.7	-
Sol-13		66.3				33.3	0.3	+
Sol-14		66.0				33.3	0.7	+
Sol-15		66.3			33.3		0.3	+
Sol-16		66.0			33.3		0.7	+
Sol-17	46.7			20.0		33.3	0.7	+
Sol-18	46.7			20.0	33.3		0.7	+
Sol-19	46.7		20.0			33.3	0.7	+
Sol-20	46.7		20.0		33.3		0.7	+
Sol-21		46.7	20.0			33.3	0.7	-
Sol-22		46.7	20.0		33.3		0.7	-
Sol-23		46.7		20.0		33.3	0.7	+
Sol-24		46.7		20.0	33.3		0.7	+

**Table 5:** The crystallinity index (%) and melting point peaks (°C) of the lipid mixtures in Table 2.

Sample	Peak (°C)	Crystallinity Index (%)
LM-1	70.48	47.09
LM-2	72.22	82.90
LM-3	67.68	20.95
LM-4	70.74	18.33
LM-5	70.28	36.84
LM-6	70.42	36.26
LM-7	67.04	23.26
LM-8	68.30	23.11
LM-9	69.94	37.92
LM-10	69.44	34.02

**Table 6:** The d(0.1), d(0.5), d(0.9), and span value of test NLCs.

Sample	d(0.1)	d(0.5)	d(0.9)	Span Value
T-NLC1	1.585	6.095	24.088	3.692
T-NLC2	0.066	0.130	0.308	1.859
T-NLC3	0.077	0.115	0.172	0.830
T-NLC4	0.071	0.121	0.210	1.140
T-NLC5	2.426	7.959	26.088	2.973
T-NLC6	0.066	0.132	0.362	2.241
T-NLC7	0.067	0.136	0.299	1.707
T-NLC8	0.075	0.120	0.192	0.975
T-NLC9	-	-	-	-
T-NLC10	-	-	-	-
T-NLC11	0.112	0.388	1.188	2.776
T-NLC12	-	-	-	-
T-NLC13	-	-	-	-
T-NLC14	-	-	-	-
T-NLC15	-	-	-	-
T-NLC16	-	-	-	-

- not measurable.

**Table 7:** The Z_ave_, PDI, zeta potential, and EE% of NLCs 1–8.

	After 1 Day				After 1 Month		
Sample	Z_ave_ (d.nm)	PDI	ZP (mV)	EE%	Z_ave_ (d.nm)	PDI	ZP (mV)
NLC1	196.47 ± 3.91	0.19 ± 0.01	−14.00 ± 0.1	93.81	194.40 ± 0.66	0.20 ± 0.01	−28.87 ± 9.90
NLC2	261.73 ± 2.44	0.18 ± 0.01	−14.43 ± 0.80	94.68	265.17 ± 1.70	0.18 ± 0.02	−21.30 ± 0.56
NLC3	182.97 ± 1.85	0.14 ± 0.01	−10.97 ± 0.25	92.66	183.20 ± 0.82	0.17 ± 0.01	−18.70 ± 0.46
NLC4	256.83 ± 0.78	0.19 ± 0.01	−13.67 ± 0.49	93.05	n.m	n.m	n.m
NLC5	94.60 ± 0.66	0.12 ± 0.00	−9.71 ± 0.15	91.89	102.60 ± 0.61	0.23 ± 0.01	−18.83 ± 2.83
NLC6	143.90 ± 1.47	0.16 ± 0.02	−10.53 ± 0.75	91.97	n.m	n.m	n.m
NLC7	92.18 ± 0.49	0.12 ± 0.02	−7.62 ± 0.26	88.31	91.38 ± 0.25	0.15 ± 0.02	−17.13 ± 0.35
NLC8	150.33 ± 2.31	0.19 ± 0.00	−8.30 ± 0.15	87.24	143.17 ± 0.57	0.19 ± 0.01	−16.40 ± 2.78

n.m: not measurable.

**Table 8:** Summary of the drug release study: the rate constant (K) and the correlation coefficient (R^2^) of the Higuchi model, the amount of the released DXM at 6 h of the release study; and the results of the statistical analysis, where the NLCs were compared with the DXM suspension.

Formulation	Higuchi Model		Released DXM at 6 h (µg)	Results of the Statistical Analysis
	K	R^2^		
DXM Suspension	3.69	0.9836	68.5 ± 4.5	reference system
NLC1	3.65	0.9967	56.7 ± 4.8	highly significant (*p* ≤ 0.001) from 0.5 h
NLC3	6.73	0.9940	104.2 ± 5.2	highly significant (*p* ≤ 0.001) from 3 h
NLC5	4.68	0.9971	76.2 ± 0.2	significant (*p* ≤ 0.05) at 6 h
NLC7	9.26	0.9968	140.5 ± 0.2	highly significant (*p* ≤ 0.001) from 1 h
